# Supplementary material for: Identification of Quantitative Trait Loci Controlling Root Morphological Traits in an Interspecific Soybean Population Using 2D Imagery Data
Source: Int J Mol Sci. 2024 Apr 25;25(9):4687. doi: 10.3390/ijms25094687 (PMC11083680; doi:10.3390/ijms25094687)
Supplement: Supplementary file 1 [file ijms-25-04687-s001.zip › ijms-2946782-supplementary.pdf]

Table S1. List of 198 putative genes with annotation function within the two most significant flanking regions of chromosomes 3 and 13 of soybean mapping populations.

Traits	Chr.	Flanking region	Gene name	Description (function)
AD<br>RV<br>LAD	03	Gm03:829023..3365988	*Glyma.03G010700*	RIBOFLAVIN BIOSYNTHESIS PROTEIN RIBS
			Glyma.03G011200	AT HOOK MOTIF DNA BINDING FAMILY PROTEIN
			Glyma.03G017900	UDPGALACTOSE/UDP GLUCOSE TRANSPORTER 2
			Glyma.03G020300	SERINE/THREONINE PROTEIN KINASE AURORA3
			Glyma.03G013700	DOWNSTREAM TARGET OF AGL15 2
			Glyma.03G011000	BR SIGNALING KINASE 3-RELATED
			Glyma.03G008600	SIGNAL PEPTIDE PEPTIDASEL IKE 4
			Glyma.03G010200	PLANT PHOSPHORIBOSYLTRANSFERASE C-TERMINAL (PRT_C)
			Glyma.03G014900	ATP-DEPENDENT RNA HELICASE DDX47-RELATED
			Glyma.03G009900	FBOX/KELCHREPEAT PROTEIN SKIP4-RELATED
			Glyma.03G013300	MYB-LIKE DNA BINDING PROTEIN MYB
			Glyma.03G021300	EPIDIDYMAL MEMBRANE PROTEIN E9-RELATED
			Glyma.03G009800	ACYLCOA THIOESTERASE
			Glyma.03G016000	BON1-ASSOCIATED PROTEIN 1-RELATED
			Glyma.03G021700	SERINE/THREONINE PROTEIN KINASE AURORA3
			Glyma.03G025900	GENOMIC DNA, CHROMOSOME 3, P1 CLONE: MDJ14-RELATED
			Glyma.03G019400	AGAMOUS-LIKE MADSBOX PROTEIN AGL13-RELATED
			Glyma.03G029800	AXIAL REGULATOR YABBY 1-RELATED
			Glyma.03G022900	PROTEIN BOI2
			Glyma.03G027000	PLANT PROTEIN OF UNKNOWN FUNCTION (DUF247)
			Glyma.03G025400	ZINC FINGER PROTEIN 183
			Glyma.03G029100	PECTINESTERASE/PECTINESTERASE INHIBITOR 17-RELATED
			Glyma.03G011900	HELITRON HELICASE-LIKE DOMAIN AT N-TERMINUS (HELITRON_LIKE_N)
			Glyma.03G025500	PROTEIN S-ACYL TRANSFERASE 13-RELATED
			Glyma.03G018300	POLYNUCLEOTIDYL TRANSFERASE, RIBONUCLEASE H-LIKE SUPERFAMILY PROTEIN
			Glyma.03G017200	ALTERNATIVE SPLICING FACTOR SRP55/B52/SRP75 (RRM SUPERFAMILY)
			Glyma.03G012000	FAS-ASSOCIATED PROTEIN
			Glyma.03G029200	PECTINESTERASE/PECTINESTERASE INHIBITOR 17-RELATED
			Glyma.03G022800	CALCIUM BINDING PROTEIN CML24-RELATED
			Glyma.03G027500	TRANSKETOLASE/GLYCOALDEHYDE TRANSFERASE
			Glyma.03G024300	CHITINASE/POLYBETAGLUCOSAMINIDASE

			Glyma.03G028900	PECTINESTERASE/PECTINESTERASE INHIBITOR 36-RELATED
			Glyma.03G028000	ARGINASE/CANAVANASE
			Glyma.03G024000	ATP-DEPENDENT RNA HELICASE DHX37-RELATED
			Glyma.03G022300	4 HYDROXYTETRAHYDRODIPICOLINATE SYNTHASE/DIHYDRODIPICOLINATE SYNTHETASE
			Glyma.03G029000	PECTINESTERASE/PECTINESTERASE INHIBITOR 17-RELATED
			Glyma.03G020800	C2 CALCIUM/LIPID BINDING ENDONUCLEASE/-EXONUCLEASE/PHOSPHATASE-RELATED
			Glyma.03G013500	ROP GUANINE NUCLEOTIDE EXCHANGE FACTOR 2-RELATED
			Glyma.03G019600	TRANSCRIPTIONAL COREPRESSOR COMPONENT
			Glyma.03G027400	3PHOSPHOSHIKIMATE 1 CARBOXYVINYLTRANSFERASE/EPSP SYNTHASE
			Glyma.03G028700	ACETYL COA-BENZYL ALCOHOL ACETYL TRANFERASE-LIKE PROTEIN-RELATED
			Glyma.03G016600	HOMEOBOX DOMAIN (HOMEOBOX)//START DOMAIN (START)
			Glyma.03G022700	ATHOOK MOTIF NUCLEAR LOCALIZED PROTEIN 18-RELATED
			Glyma.03G012200	PROTEIN SAY1
			Glyma.03G026600	PROTEIN OF UNKNOWN FUNCTION
			Glyma.03G030100	CYTOCHROME P450 71B21-RELATED
			Glyma.03G027200	PROTEIN KINASE DOMAIN (PKINASE)//LEUCINE RICH REPEAT (LRR_8)
			Glyma.03G017100	ARMADILLO/BETACATENIN REPEAT CONTAINING PROTEIN-RELATED
AD			*Glyma.03G016200*	BON1-ASSOCIATED PROTEIN 1-RELATED
RV			*Glyma.03G012800*	KDEL LYSASPGLULEU CONTAINING-RELATED
LAD			*Glyma.03G027800*	PPR REPEAT (PPR)//PPR REPEAT FAMILY (PPR_2)
			Glyma.03G018500	ARSENITE-TRANSPORTING ATPASE/ARSENITE-TRANSLOCATING ATPASE
			Glyma.03G024500	CHITINASE/POLYBETAGLUCOSAMINIDASE
			Glyma.03G024700	SULFATE TRANSPORTER 3.3-RELATED
			Glyma.03G028400	PLANT PROTEIN OF UNKNOWN FUNCTION (DUF641) (DUF641)
			Glyma.03G026400	EXOCYST COMPLEX COMPONENT SEC6-RELATED
			Glyma.03G013600	ADENOSINE-DEAMINASE (EDITASE) DOMAIN (A_DEAMIN)
			Glyma.03G021200	GERANIOL 8 HYDROXYLASE/CYTOCHROME P450, FAMILY 76, SUBFAMILY C, POLYPEPTIDE 4
			Glyma.03G029600	AUXIN REGULATED PROTEIN-RELATED
			Glyma.03G015400	EXTENDED SYNAPTOTAGMIN-RELATED
			Glyma.03G023700	PROTEIN KINASE DOMAIN (PKINASE)//LEUCINE RICH REPEAT (LRR_1)//LEUCINE RICH REPEAT (LRR_8)

			Glyma.03G014500	DEHYDROGENASES WITH DIFFERENT SPECIFICITIES (RELATED TO SHORT CHAIN ALCOHOL DEHYDROGENASES)
			Glyma.03G019300	MADSBOX PROTEIN SOC1
			Glyma.03G009400	PREDICTED CA^{2+}-DEPENDENT PHOSPHOLIPID BINDING PROTEIN
			Glyma.03G014800	AUXIN RESPONSIVE PROTEIN (AUXIN_INDUCIBLE)
			Glyma.03G013100	MITOCHONDRIAL RIBOSOMAL PROTEIN S33
			Glyma.03G012900	KDEL LYSASPGLULEU CONTAINING-RELATED
			Glyma.03G026100	EXOCYST COMPLEX COMPONENT 3
			Glyma.03G026700	PPR REPEAT (PPR)//PPR REPEAT FAMILY (PPR_2)
			Glyma.03G019500	PROTEIN Y45F10A.7, ISOFORM A
			Glyma.03G013400	REMORIN, NTERMINAL REGION (REMORIN_N)
			Glyma.03G020400	CYTOCHROME P450, FAMILY 704, SUBFAMILY A, POLYPEPTIDE 1
			Glyma.03G028800	METHIONINE AMINOPEPTIDASE 1
			Glyma.03G025800	GENOMIC DNA, CHROMOSOME 3, P1 CLONE: MDJ14-RELATED
			Glyma.03G009200	MEDIATOR OF RNA POLYMERASE II TRANSCRIPTION SUBUNIT 12
			Glyma.03G015700	FAST LEU-RICH DOMAIN CONTAINING
			Glyma.03G023500	AT HOOK MOTIF DNA BINDING FAMILY PROTEIN
			Glyma.03G020100	EXPRESSED PROTEIN
			Glyma.03G027900	PREDICTED METALLOPROTEASE WITH CHAPERONE ACTIVITY (RNASE H/HSP70 FOLD)
			Glyma.03G021100	RIBOSOMAL RNA PROCESSING PROTEIN 36 HOMOLOG
			Glyma.03G018200	POLYNUCLEOTIDYL TRANSFERASE, RIBONUCLEASE H-LIKE SUPERFAMILY PROTEIN
			Glyma.03G016400	BON1-ASSOCIATED PROTEIN 1-RELATED
			Glyma.03G016700	METHYLTRANSFERASE PMT13-RELATED
			Glyma.03G019100	SUBTILASE FAMILY PROTEIN-RELATED
			Glyma.03G022200	LITTLE ZIPPER 1 PROTEIN-RELATED
			Glyma.03G029900	CYTOCHROME P450 71B21-RELATED
			Glyma.03G015100	DTDP4DEHYDRORHAMNOSE REDUCTASE
			Glyma.03G025300	ABSCISIC ACID RECEPTOR PYL10-RELATED
			Glyma.03G009500	TRANSCRIPTION FACTOR MYC1
			Glyma.03G016500	SHAGGY-RELATED PROTEIN KINASE BETA-RELATED
			Glyma.03G027600	DNA REPAIR PROTEIN RAD51 HOMOLOG 3
			Glyma.03G019800	GIBBERELLIN 20 OXIDASE 1-RELATED
			Glyma.03G029300	ENTH/VHS/GAT FAMILY PROTEIN
			Glyma.03G015600	CELL DIVISION PROTEIN FTSY HOMOLOG, CHLOROPLASTIC
			Glyma.03G017500	RING FINGER DOMAIN CONTAINING
			Glyma.03G023100	LOB DOMAIN CONTAINING PROTEIN 19-RELATED
			Glyma.03G021900	GROWTH REGULATING FACTOR 9

			Glyma.03G021800	NADHUBIQUINONE OXIDOREDUCTASE
			Glyma.03G017000	FBOX PROTEIN PP2A13
			Glyma.03G020500	MEMBER OF "GDXG" FAMILY OF LIPOLYTIC ENZYMES
AD<br>RV<br>LAD	13	Gm13:27527083..43496306	*Glyma.13G175000*	DUF679 DOMAIN MEMBRANE PROTEIN 7
			Glyma.13G310900	RING ZINC FINGER PROTEIN
			Glyma.13G172800	RAS-RELATED PROTEIN RABA1G
			Glyma.13G171200	RIBOSOMAL RNA PROCESSING PROTEIN 7-RELATED
			Glyma.13G165200	40S RIBOSOMAL PROTEIN S11 FAMILY MEMBER
			Glyma.13G177300	MITOCHONDRIAL ARGININE TRANSPORTER BAC1
			Glyma.13G159900	SMALL NUCLEAR RNA ACTIVATING COMPLEX, POLYPEPTIDE 1
			Glyma.13G226900	PROTEIN OF UNKNOWN FUNCTION (DUF1685) (DUF1685)
			Glyma.13G172200	APOPTOSIS INHIBITOR 5
			Glyma.13G175600	DROUGHT INDUCED 19 PROTEIN (DI19), ZINC BINDING
			Glyma.13G162300	UBIQUITIN SPECIFIC PROTEASE
			Glyma.13G178200	NON-SENSE-MEDIATED mRNA DECAY PROTEIN
			Glyma.13G173500	2HYDROXYISOFLAVANONE SYNTHASE/ISOFLAVONOID SYNTHASE
			Glyma.13G172700	HOMEOBOX DOMAIN (HOMEOBOX)
			Glyma.13G173600	O-METHYL TRANSFERASE
			Glyma.13G170700	POTASSIUM TRANSPORTER 8
			Glyma.13G161400	XYLOGALACTURONAN BETA-1,3-XYLOSYLTRANSFERASE
			Glyma.13G167600	SOLUTE CARRIER FAMILY 35
			Glyma.13G167500	GLYCOSYLTRANSFERASE 14 FAMILY MEMBER
			Glyma.13G162500	ARP2/3 COMPLEX 21 KD SUBUNIT
			Glyma.13G176400	POLYUBIQUITIN 3
			Glyma.13G168600	PROTEIN FARNESYLTRANSFERASE SUBUNIT BETA
			Glyma.13G160400	HYDROPHOBIC SEED PROTEIN (HYDROPHOB_SEED)
			Glyma.13G166000	HISTON ELYSINE N-METHYL TRANSFERASE, H3 LYSINE9 SP
			Glyma.13G173100	HISTONE H3
			Glyma.13G167100	PEROXIDASE 25
			Glyma.13G167900	RIBOSOME BIOGENESIS REGULATORY PROTEIN
			Glyma.13G196900	DNA DIRECTED RNA POLYMERASE V SUBUNIT 1
			Glyma.13G168800	PLASTOCYANIN-LIKE DOMAIN (CU_BIND_LIKE)
			Glyma.13G175100	SOLUTE CARRIER FAMILY 35
			Glyma.13G224000	MOLECULAR CHAPERONES GRP78/BIP/KAR2, HSP70 SUPERFAMILY
			Glyma.13G165700	EQUILIBRATIVE NUCLEOTIDE TRANSPORTER 8
			Glyma.13G174800	PROTEIN KINASE FAMILY PROTEIN

			Glyma.13G166200	FBOX-LIKE (FBOX-LIKE)//LEUCINE-RICH REPEAT
			Glyma.13G265400	RING BOX
			Glyma.13G244500	PROTEIN OF UNKNOWN FUNCTION (DUF707) (DUF707)
			Glyma.13G174900	RECEPTORL IKE PROTEIN KINASE HSL1
			Glyma.13G268600	PLUG DOMAIN OF SEC61P (PLUG_TRANSLOCON)
			Glyma.13G159800	BED FINGER-RELATED
			Glyma.13G169000	CALCIUM HOMEOSTASIS REGULATOR-RELATED
			Glyma.13G174300	XBOX TRANSCRIPTION FACTOR-RELATED
			Glyma.13G168700	2HYDROXYACID DEHYDROGENASE-RELATED
			Glyma.13G190400	LEUCINE-RICH REPEAT CONTAINING PROTEIN
			Glyma.13G176700	PROTEASOME SUBUNIT ALPHA TYPE1
			Glyma.13G172000	IQDOMAIN 9 PROTEIN
			Glyma.13G172500	ZINC FINGER FYVE DOMAIN CONTAINING PROT
			Glyma.13G169700	BIDIRECTIONAL SUGAR TRANSPORTER SWEET4-RELATED
			Glyma.13G165500	RING FINGER DOMAIN CONTAINING
			Glyma.13G336600	EXPANSINA6
			Glyma.13G337300	SCARECROW-LIKE PROTEIN 14-RELATED
			Glyma.13G330900	CHITINASE
			Glyma.13G332000	COPPER TRANSPORT PROTEIN ATOX1-RELATED
			Glyma.13G328800	NODULIN (NODULIN)
			Glyma.13G337100	PROTEIN TBF1
			Glyma.13G338100	MULTICOPPER OXIDASE
			Glyma.13G329500	PROTEIN NRT1/PTR FAMILY 2.1-RELATED
			Glyma.13G334200	RNA AND EXPORT FACTOR BINDING PROTEIN
			Glyma.13G329000	DOF ZINC FINGER PROTEIN DOF1.1-RELATED
			Glyma.13G338300	ZINC/IRON TRANSPORTER
			Glyma.13G343600	PROTEIN FARNESYLTRANSFERASE/FTASE//PROTEINGERANYL GERANYL-TRANSFERASE TYPE I
			Glyma.13G335600	ACTIN
			Glyma.13G338000	DVL13-RELATED
			Glyma.13G335900	PHOSPHATIDYLINOSITOL NACETYL-GLUCOSAMINYL TRANSFERASE SUBUNIT P DOWN SYNDROME CRITICAL REGION PROTEIN 5-RELATED
			Glyma.13G337500	SCARECROW-LIKE PROTEIN 11-RELATED
			Glyma.13G329700	ETHYLENE RESPONSIVE TRANSCRIPTION FACTOR RAP27
			Glyma.13G330400	FLIPPASE KINASE 1-RELATED
			Glyma.13G341500	DNADAMAGEREPAIR/TOLERATION PROTEIN DRT100-RELATED
			Glyma.13G336100	OUTER ENVELOPE PORE PROTEIN 161, CHLOROPLASTIC
			Glyma.13G331300	RECEPTORL IKE PROTEIN KINASE HERK 1

			Glyma.13G330600	KINESIN MOTOR PROTEIN-RELATED
			Glyma.13G334300	HISTONE 2A
			Glyma.13G332700	60S RIBOSOMAL PROTEIN L23A
			Glyma.13G331700	EXOCYST COMPLEX PROTEIN EXO70
			Glyma.13G330300	RING FRINGE-RELATED
			Glyma.13G338700	PROTEIN TOC753, CHLOROPLASTIC-RELATED
			Glyma.13G333300	METHYLCPGBINDING DOMAIN CONTAINING PROTEIN 1-RELATED
			Glyma.13G342700	ANTHRANILATE PHOSPHORIBOSYL TRANSFERASE-LIKE PROTEIN
			Glyma.13G338200	ZINC/IRON TRANSPORTER
			Glyma.13G333200	TRANSCRIPTION FACTOR MYB48-RELATED
			Glyma.13G342500	ETHYLENE INSENSITIVE 3-LIKE 3 PROTEIN
			Glyma.13G341600	PROTEIN FAR1-RELATED SEQUENCE 4
			Glyma.13G331900	60S RIBOSOMAL PROTEIN L35
			Glyma.13G330800	CHITINASE
			Glyma.13G333800	HISTONE 2A
			Glyma.13G339700	MITOCHONDRIAL IMPORT INNER MEMBRANE TRANSLOCASE SUBUNIT TIM10
			Glyma.13G341100	PROTEIN TYROSINE KINASE (PKINASE_TYR)//TPR REPEAT (TPR_11)
			Glyma.13G338400	RING ZINC FINGER PROTEIN
			Glyma.13G338600	DNAJ HOMOLOG SUBFAMILY C MEMBER
			Glyma.13G342000	HISTONE H3
			Glyma.13G340400	E3 UBIQUITIN LIGASE BIG BROTHER
			Glyma.13G340900	PROTEIN ZNTB
			Glyma.13G335300	E3 UBIQUITINPROTEIN LIGASE XBAT31-RELATED
			Glyma.13G333100	AQUAPORIN TRANSPORTER
			Glyma.13G333900	HISTONE 2A
			Glyma.13G341400	AGC (CAMP-DEPENDENT, CGMP-DEPEN-DENT AND PROTEIN KINASE C) KINASE FAMILY PROTEIN
			Glyma.13G343000	GLYCOSYL HYDROLASE
			Glyma.13G340100	ORIGIN RECOGNITION COMPLEX SUBUNIT 1

Table S2. List of SNP variations between 'William 82' and 'PI366121' along with the genes underlying the most significant root QTL genomic regions.

Position	Gene	Ref	ALT	Effect	Start	End	Strand
Chr03:1040647	*Glyma.03G010700*	A	T	3_prime_UTR_variant	1,037,842	1,042,141	+
Chr03:1789064	*Glyma.03G017900*	T	C	Upstream gene variant	1,787,790	1,800,965	+
Chr03:2075749	*Glyma.03G020300*	G	T/A	Upstream gene variant	2,074,821	2,078,561	-
Chr03:1393538	*Glyma.03G013700*	G	T	Missense variant	1,393,484	1,393,734	+
Chr03:831779	*Glyma.03G008600*	C	G	Downstream gene variant	830,451	838,408	-
Chr03:1496887	*Glyma.03G014900*	G	C	Downstream gene variant	1,494,708	1,502,910	-
Chr03:2805098	*Glyma.03G025900*	G	A	Synonymous variant	2,804,898	2,805,383	+
Chr03:1981263	*Glyma.03G019400*	A	G	Intron variant	1,977,661	1,985,026	-
Chr03:2413616	*Glyma.03G022900*	C	T	Missense variant	2,413,172	2,413,671	-
Chr03:1738562	*Glyma.03G017200*	G	A	5_prime_UTR_variant	1,735,158	1,738,810	-
Chr03:3186369	*Glyma.03G029200*	T	G	Synonymous variant	3,185,979	3,188,159	-
Chr03:3022150	*Glyma.03G027500*	C	T	Synonymous variant	3,022,087	3,022,240	-
Chr03:3151051	*Glyma.03G028900*	G	A	Intron variant	3,148,672	3,152,887	-
Chr03:3072041	*Glyma.03G028000*	G	C	Missense variant	3,071,857	3,072,066	-
Chr03:3165706	*Glyma.03G029000*	C	T	Intron variant	3,164,736	3,166,769	+
Chr03:2124946	*Glyma.03G020800*	A	G	3_prime_UTR_variant	2,124,895	2,135,692	-
Chr03:2009608	*Glyma.03G019600*	G	C	3_prime_UTR_variant	2,007,617	2,009,871	+
Chr03:3010747	*Glyma.03G027400*	C	T	Missense variant	3,010,213	3,018,498	-
Chr03:3128983	*Glyma.03G028700*	A	G	Missense variant	3,128,776	3,130,648	+
Chr03:2998840	*Glyma.03G027200*	A	G	Missense variant	2,997,332	2,999,026	+
Chr03:1295079	*Glyma.03G012800*	A	G	Intron variant	1,292,400	1,298,429	-
Chr03:1852211	*Glyma.03G018500*	A	C	Intron variant	1,849,093	1,855,316	+
Chr03:3109886	*Glyma.03G028400*	T	C	Missense variant	3,107,378	3,110,786	+
Chr03:2902943	*Glyma.03G026400*	C	G	Intron variant	2,892,021	2,904,360	+
Chr03:1385095	*Glyma.03G013600*	CA	C	Intron variant	1,379,993	1,390,392	+
Chr03:2171360	*Glyma.03G021200*	G	A	Downstream gene variant	2,170,567	2,172,891	-
Chr03:3253737	*Glyma.03G029600*	T	C	3_prime_UTR_variant	3,253,478	3,255,163	-
Chr03:1452530	*Glyma.03G014500*	C	T	Missense variant	1,452,354	1,452,677	-
Chr03:2490109	*Glyma.03G023500*	C	A	Intron variant	2,486,847	2,493,916	+
Chr03:2061229	*Glyma.03G020100*	A	C	Missense variant	2,057,797	2,063,029	-
Chr03:3053137	*Glyma.03G027900*	T	C	Intron variant	3,048,805	3,061,575	-
Chr03:2164039	*Glyma.03G021100*	C	T	Splice region variant	2,162,406	2,167,712	+
Chr03:3029835	*Glyma.03G027600*	A	G	Splice region variant	3,029,154	3,033,239	-
Chr03:2043655	*Glyma.03G019800*	T	G	Synonymous variant	2,043,161	2,043,723	+
Chr03:3225850	*Glyma.03G029300*	T	C	Synonymous variant	3,225,772	3,226,185	-
Chr03:1547374	*Glyma.03G015600*	A	G	Upstream gene variant	1,539,775	1,548,226	-
Chr03:2286654	*Glyma.03G021900*	G	T	Intron variant	2,285,871	2,289,619	-
Chr03:2092471	*Glyma.03G020500*	G	A	Intron variant	2,091,153	2,093,117	+
Chr13:28686299	*Glyma.13G172800*	G	C	Missense variant	28,683,493	28,686,774	+
Chr13:28537151	*Glyma.13G171200*	T	C	Intron variant	28,536,983	28,540,336	-
Chr13:29123039	*Glyma.13G177300*	C	A	5_prime_UTR_variant	29,122,910	29,128,171	+
Chr13:27561946	*Glyma.13G159900*	T	C	Upstream gene variant	27,560,735	27,566,769	+
Chr13:28613474	*Glyma.13G172200*	A	G	Downstream gene variant	28,612,286	28,620,441	-
Chr13:27774334	*Glyma.13G162300*	G	T	3_prime_UTR_variant	27,773,991	27,778,719	-
Chr13:28672639	*Glyma.13G172700*	T	A	Missense variant	28,671,831	28,675,231	+

Chr13:28488019	*Glyma.13G170700*	G	C	5_prime_UTR_variant	28,487,838	28,494,611	+
Chr13:27700711	*Glyma.13G161400*	C	T	Downstream gene variant	27,698,462	27,701,365	-;-
Chr13:28211158	*Glyma.13G167600*	T	G	Synonymous variant	28,210,440	28,212,300	+
Chr13:27786625	*Glyma.13G162500*	A	T	Upstream gene variant	27,785,225	27,788,045	-
Chr13:28303515	*Glyma.13G168600*	G	A	Missense variant	28,298,272	28,304,199	-
Chr13:27606195	*Glyma.13G160400*	C	T	3_prime_UTR_variant	27,606,020	27,608,359	-
Chr13:28720934	*Glyma.13G173100*	C	T	Synonymous variant	28,720,656	28,721,572	+
Chr13:28182349	*Glyma.13G167100*	T	A	Missense variant	28,182,121	28,182,399	+
Chr13:28240930	*Glyma.13G167900*	T	G	Upstream gene variant	28,240,381	28,243,803	+
Chr13:31057839	*Glyma.13G196900*	T	G	Downstream gene variant	31,055,891	31,072,513	+
Chr13:28317204	*Glyma.13G168800*	C	T	Upstream gene variant	28,316,886	28,317,715	+
Chr13:28970569	*Glyma.13G175100*	A	C	Downstream gene variant	28,969,904	28,975,782	+
Chr13:33685460	*Glyma.13G224000*	T	A	Missense variant	33,684,833	33,686,902	+
Chr13:28928217	*Glyma.13G174800*	A	G	Synonymous variant	28,927,266	28,928,267	-
Chr13:28096911	*Glyma.13G166200*	G	A	Intron variant	28,093,807	28,097,420	-
Chr13:35388368	*Glyma.13G244500*	T	A	Upstream gene variant	35,387,923	35,395,610	+
Chr13:28938817	*Glyma.13G174900*	A	C	Synonymous variant	28,938,189	28,940,823	-
Chr13:28305241	*Glyma.13G168700*	C	T	Splice donor variant	28,305,098	28,310,222	+
Chr13:30403159	*Glyma.13G190400*	G	T	Missense variant	30,402,029	30,409,606	+
Chr13:28595304	*Glyma.13G172000*	T	C	Upstream gene variant	28,593,222	28,598,072	-
Chr13:28645033	*Glyma.13G172500*	G	A	Synonymous variant	28,644,819	28,649,188	-
Chr13:28367052	*Glyma.13G169700*	A	T	Intron variant	28,363,094	28,367,579	-
Chr13:28031079	*Glyma.13G165500*	A	T	Downstream gene variant	28,030,788	28,034,710	+
Chr13:42993020	*Glyma.13G336600*	A	G	Synonymous variant	42,991,934	42,993,435	-
Chr13:42797618	*Glyma.13G334200*	T	C	Missense variant	42,794,878	42,801,645	+
Chr13:42918068	*Glyma.13G335600*	T	A	Synonymous variant	42,916,206	42,918,785	+
Chr13:42423055	*Glyma.13G329700*	C	T	Intron variant	42,422,213	42,426,774	-
Chr13:42503906	*Glyma.13G330400*	A	G	Intron variant	42,494,484	42,509,495	+
Chr13:43326463	*Glyma.13G341500*	C	A	Missense variant	43,325,404	43,326,510	-
Chr13:42581208	*Glyma.13G331300*	A	T	Synonymous variant	42,580,671	42,582,344	-
Chr13:42523190	*Glyma.13G330600*	A	T	Missense variant	42,523,075	42,523,280	-
Chr13:42807826	*Glyma.13G334300*	A	T	Synonymous variant	42,807,791	42,808,000	+
Chr13:42687849	*Glyma.13G332700*	C	T	5_prime_UTR_variant	42,687,782	42,689,387	+
Chr13:42740451	*Glyma.13G333300*	A	T	Synonymous variant	42,739,408	42,742,467	-
Chr13:42713462	*Glyma.13G333200*	C	A	Synonymous variant	42,713,045	42,714,681	-
Chr13:43333456	*Glyma.13G341600*	T	C	Missense variant	43,331,375	43,335,735	-
Chr13:42650050	*Glyma.13G331900*	A	G	Splice region variant	42,648,871	42,650,712	-
Chr13:43295111	*Glyma.13G341100*	C	A	Downstream gene variant	43,292,117	43,296,459	-
Chr13:43246090	*Glyma.13G340400*	G	A	Splice region variant	43,244,791	43,246,867	-
Chr13:43284804	*Glyma.13G340900*	A	G	Upstream gene variant	43,282,672	43,286,972	-
Chr13:42711332	*Glyma.13G333100*	G	A	Missense variant	42,711,023	42,711,397	+
Chr13:43311794	*Glyma.13G341400*	C	A	Missense variant	43,311,148	43,312,308	+
Chr13:43447450	*Glyma.13G343000*	G	C	Missense variant	43,447,390	43,451,125	-
Chr13:43217945	*Glyma.13G340100*	T	A	3_prime_UTR_variant	43,210,673	43,218,069	+

Table S3. List of candidate genes showing high expression in the root with annotation descriptions.

GENE NAME	ANNOTATION DESCRIPTION
Glyma.03G013700	DOWNSTREAM TARGET OF AGL15 2
Glyma.03G008600	SIGNAL PEPTIDE PEPTIDASE-LIKE 4
Glyma.03G022900	GUANYL-NUCLEOTIDE EXCHANGE FACTOR
Glyma.03G029100	PHOSPHOENOL-PYRUVATE DIKINASE-RELATED
Glyma.03G027500	TRANSKETOLASE/GLYCOALDEHYDE TRANSFERASE
Glyma.03G028000	ARGINASE/CANAVANASE
Glyma.03G027400	3PHOSPHOSHIKIMATE 1CARBOXYVINYLTRANSFERASE/EPSP SYNTHASE
Glyma.03G028700	TRANSFERASE ACTIVITY, TRANSFERRING ACYL GROUPS OTHER THAN AMINO-ACYL GROUPS
Glyma.03G027200	LEUCINE-RICH REPEAT RECEPTOR-LIKE PROTEIN KINASE
Glyma.03G027800	PPR REPEAT (PPR)//PPR REPEAT FAMILY (PPR_2)
Glyma.03G028400	PLANT PROTEIN OF UNKNOWN FUNCTION (DUF641) (DUF641)
Glyma.03G014500	DEHYDROGENASES WITH DIFFERENT SPECIFICITIES (RELATED TO SHORTCHAIN ALCOHOL DEHYDROGENASES)
Glyma.03G020100	NUCLEOTIDYLTRANSFERASE FAMILY PROTEIN
Glyma.03G021100	RIBOSOMAL RNA PROCESSING PROTEIN 36 HOMOLOG
Glyma.03G027600	DNA REPAIR PROTEIN RAD51 HOMOLOG 3
Glyma.13G172800	GTPASE RAB11/YPT3, SMALL G PROTEIN SUPERFAMILY
Glyma.13G172700	HOMEOBOX DOMAIN (HOMEOBOX)
Glyma.13G168600	PROTEIN FARNESYLTRANSFERASE SUBUNIT BETA
Glyma.13G167100	PEROXIDASE 25
Glyma.13G224000	HEAT SHOCK PROTEIN 70 KDA
Glyma.13G168700	2 HYDROXY-ACID DEHYDROGENASE-RELATED
Glyma.13G190400	LEUCINE RICH REPEAT CONTAINING PROTEIN
Glyma.13G172500	ZINC FINGER FYVE DOMAIN CONTAINING PROTEIN
Glyma.13G334200	RNA AND EXPORT FACTOR BINDING PROTEIN
Glyma.13G341500	LEUCINE-RICH REPEAT RECEPTOR-LIKE PROTEIN KINASE
Glyma.13G330600	KINESIN MOTOR PROTEIN-RELATED
Glyma.13G341600	PROTEIN FAR1-RELATED SEQUENCE 4
Glyma.13G331900	60S RIBOSOMAL PROTEIN L35
Glyma.13G333100	AQUAPORIN TRANSPORTER
Glyma.13G341400	AGC (cAMP-dependent, cGMP-dependent and protein kinase C) KINASE FAMILY PROTEIN
Glyma.13G343000	GLYCOSYL HYDROLASE
Glyma.13G340400	RING FINGER DOMAIN-CONTAINING

Table S4. List of candidate genes with various tissue/organ-specific values in soybean from the ePlant soybean transcriptome data.

Gene name	Root	Root tip	Root stripped	Root hair (24HAI)	Leaves	SAM	Flower	Green pods	Nodules
Glyma.03G008600	10.02	5.56	10.11	7.50	15.63	16.72	21.05	22.55	2.39
Glyma.03G029100	1.85	0.00	0.52	0.56	0.00	0.76	0.00	0.00	0.27
Glyma.03G028000	1.58	1.55	382.18	156.61	2.13	52.94	4.74	0.68	0.00
Glyma.03G028700	2.37	0.31	0.78	0.00	0.36	1.77	0.30	0.00	0.00
Glyma.03G027200	0.00	0.00	0.00	0.00	0.36	0.00	0.00	0.00	0.00
Glyma.03G020100	34.28	19.78	23.32	16.66	24.86	52.43	48.63	32.80	50.80
Glyma.03G021100	4.48	5.25	1.04	5.28	4.97	7.35	4.45	1.37	5.05
Glyma.03G027600	4.48	8.03	5.44	4.72	7.81	8.87	6.52	6.83	1.86
Glyma.03G022900	10.81	37.39	14.77	10.00	5.33	29.38	6.52	15.03	0.80
Glyma.03G027500	239.20	364.97	164.79	170.49	27.70	136.53	37.36	225.48	57.71
Glyma.03G027400	51.95	36.78	51.56	44.43	17.40	40.78	9.19	40.31	15.69
Glyma.03G028400	17.41	14.52	18.14	14.72	10.65	19.00	11.86	14.35	15.96
Glyma.03G014500	13.98	9.89	4.40	8.89	1.07	0.76	0.30	0.68	0.80
Glyma.13G172800	2.64	5.56	3.89	5.55	1.07	2.53	3.56	2.73	2.13
Glyma.13G172700	0.00	0.00	0.00	0.28	0.00	0.00	0.00	0.68	0.00
Glyma.13G168600	8.44	17.61	19.69	8.89	9.23	16.97	10.67	13.67	16.49
Glyma.13G167100	1.85	0.00	2.33	0.28	4.26	0.25	2.08	1.37	0.00
Glyma.13G168700	32.70	3.71	25.65	21.94	18.47	34.45	265.97	210.45	4.26
Glyma.13G190400	2.11	3.40	0.52	4.17	2.13	3.29	1.78	1.37	1.60
Glyma.13G172500	2.64	3.09	0.78	3.05	20.24	0.00	1.48	0.00	0.27
Glyma.13G341500	10.02	202.42	364.56	36.37	125.36	44.07	423.42	101.13	0.27
Glyma.13G330600	9.23	33.99	7.00	10.83	13.85	30.90	11.56	11.62	41.22
Glyma.13G341600	3.96	6.80	5.70	7.22	7.81	6.84	9.19	4.10	5.58
Glyma.13G333100	61.18	10.82	14.25	21.66	12.78	78.52	37.66	14.35	2.13
Glyma.13G341400	27.95	10.82	4.66	49.15	2.84	2.79	13.05	5.47	5.05
Glyma.13G343000	0.26	0.00	0.00	5.55	0.71	91.95	3.85	0.68	0.00
Glyma.13G334200	117.62	266.39	115.04	112.73	94.82	155.28	136.69	110.69	58.77
Glyma.13G331900	14.77	43.57	27.98	30.27	7.46	17.98	2.37	8.88	6.91
Glyma.13G224000	134.50	0.93	155.46	12.77	64.63	68.39	30.84	211.82	50.53

Table S5. Distribution of SNP markers mapped on soybean chromosome/linkage groups

Chromosome	Linkage group	No. of markers	Distance (cM)	Density SNP/cM
1	D1a	56	188.56	3.37
2	D1b	88	293.83	3.33
3	N	61	246.62	4.04
4	C1	65	226.36	3.48
5	A1	71	220.26	3.10

6	C2	62	211.06	3.40
7	M	83	233.44	2.81
8	A2	81	262.07	3.24
9	K	65	212.68	3.27
10	O	78	215.62	2.76
11	B1	50	219.89	4.40
12	H	59	281.99	4.78
13	F	96	224.18	2.34
14	B2	70	172.16	2.46
15	E	71	198.24	2.79
16	J	63	180.02	2.86
17	D2	62	233.71	3.77
18	G	86	198.24	2.30
19	L	82	240.27	2.93
20	I	59	167.50	2.84
Total		1408	4426.70	3.14
Average marker and distance/chromosome		70.4	221.33	3.14

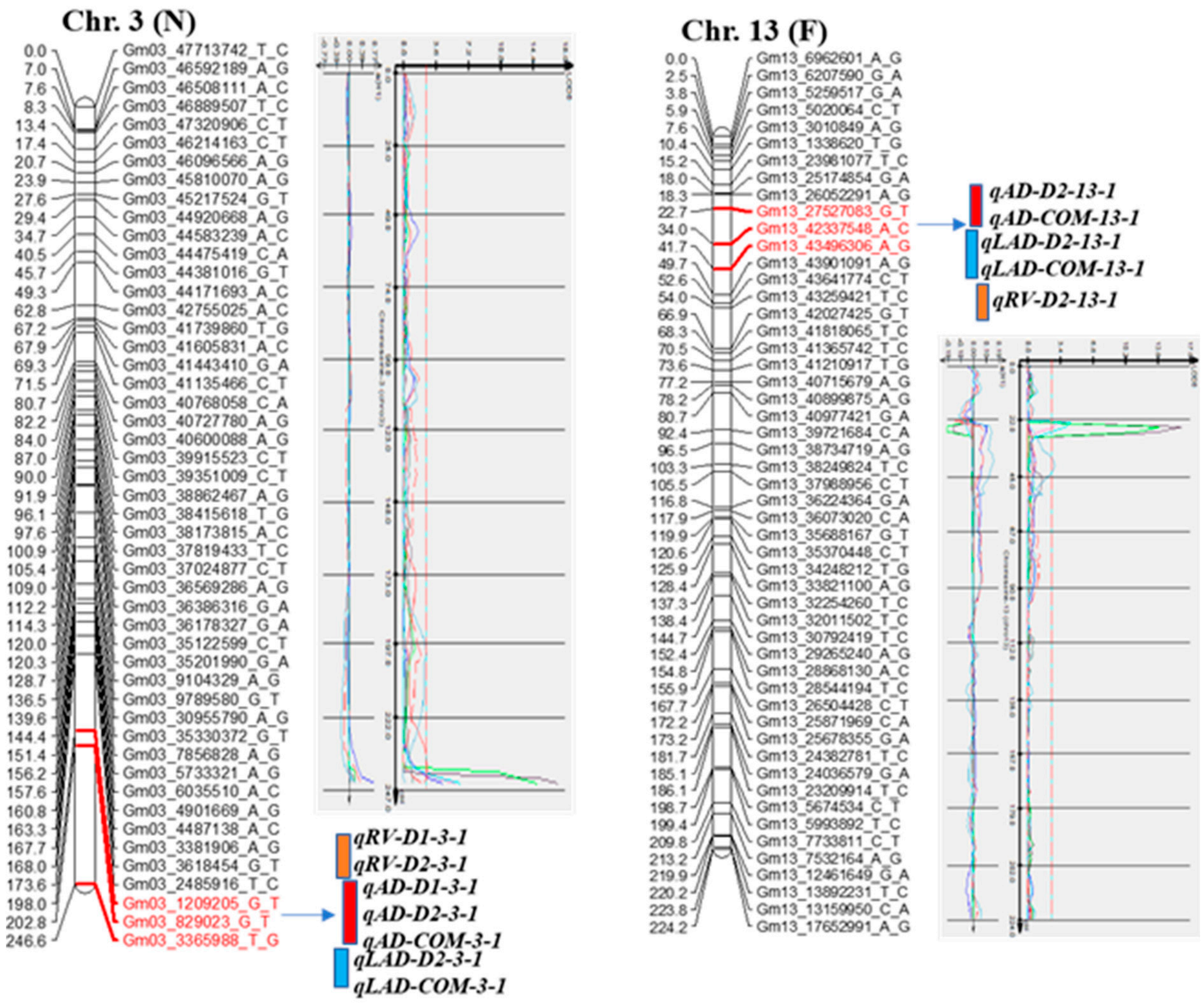

Figure S1. The most significant QTL regions with LOD distribution curve on chromosomes 3 and 13. Chr:

chromosome; AD: average diameter; RV: root volume; LAD: link average diameter. D1, D2, and COM indicate environment 1, environment 2, and combined environments, respectively. Different colors indicate different QTLs within same region.

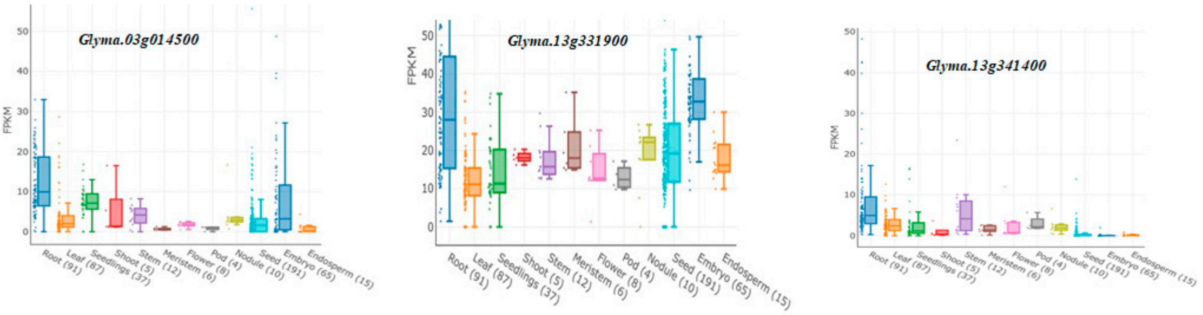

Figure S2. Expression patterns of three candidate genes across twelve different soybean tissues/organs (web-based publicly available RNA-Seq soybean libraries '4085') (http://ipf.sustech.edu.-cn/pub/soybean/). FPKM: fragments per kilobase of transcript per million mapped reads. The blue colored bar on the left indicates the expression level in the roots.
